# The Prediction of Metastases of Lateral Cervical Lymph Node in Medullary Thyroid Carcinoma

**DOI:** 10.3389/fendo.2021.741289

**Published:** 2021-11-17

**Authors:** Tian-Han Zhou, Ling-Qian Zhao, Yu Zhang, Fan Wu, Kai-Ning Lu, Lin-Lin Mao, Ke-Cheng Jiang, Ding-Cun Luo

**Affiliations:** ^1^ The Fourth School of Clinical Medicine, Zhejiang Chinese Medical University, Hangzhou, China; ^2^ Department of Surgical Oncology, Affiliated Hangzhou First People’s Hospital, Zhejiang University School of Medicine, Hangzhou, China

**Keywords:** lateral lymph node metastasis, nomogram, medullary thyroid carcinoma, SEER, prediction

## Abstract

**Purpose:**

Development and validation of a nomogram for the prediction of lateral lymph node metastasis (LLNM) in medullary thyroid carcinoma (MTC).

**Methods:**

We retrospectively reviewed the clinical features of patients with MTC in the Surveillance, Epidemiology, and End Results (SEER) database between 2010 and 2017 and in our Department of Surgical Oncology, Hangzhou First People’s Hospital between 2009 and 2019. The log‐rank test was used to compare the difference in the Kaplan–Meier (K–M) curves in recurrence and survival. The nomogram was developed to predict the risk of LLNM in MTC patients. The prediction efficiency of the predictive model was assessed by area under the curve (AUC) and concordance index (C-index) and calibration curves. Decision curve analysis (DCA) was performed to determine the clinic value of the predictive model.

**Result:**

A total of 714 patients in the SEER database and 35 patients in our department were enrolled in our study. Patients with LLNM had worse recurrence rate and cancer-specific survival (CSS) compared with patients without LLNM. Five clinical characteristics including sex, tumor size, multifocality, extrathyroidal extension, and distant metastasis were identified to be associated with LLNM in MTC patients, which were used to develop a nomogram. Our prediction model had satisfied discrimination with a C-index of 0.825, supported by both training set and internal testing set with a C-index of 0.825, and 0.816, respectively. DCA was further made to evaluate the clinical utility of this nomogram for predicting LLNM.

**Conclusions:**

Male sex, tumor size >38mm, multifocality, extrathyroidal extension, and distant metastasis in MTC patients were significant risk factors for predicting LLNM.

## Introduction

In recent years, thyroid carcinoma has exhibited the rapidest increase in incidence among solid tumors (1). Thyroid cancer can be classified into papillary thyroid cancer, follicular thyroid cancer, anaplastic thyroid cancer, and medullary thyroid carcinoma (MTC), and MTC accounts for only 1.6% in thyroid cancer (2). The American Thyroid Association (ATA) recommended that the curative therapy for MTC is primary tumor resection and cervical lymph node dissection (3). However, the extent of initial surgery for cervical lymph node dissection is controversial, in particular, whether lateral lymph node dissection (LLND) in MTC is needed. Some surgeons prefer more aggressive management to perform LLND or prophylactic dissection to improve postoperative biochemical recovery rates, whereas others advocate for conservative surgical management for possibility of complications and its unknown benefits (4, 5).

Currently, there is no accurate preoperative assessment to evaluate the lateral lymph node metastasis (LLNM) (6). If we can effectively identify who are prone to be LLNM more effectively, patients can be given more comprehensive and targeted preoperative examination and more active surgical intervention. On the one hand, it may reduce the risk of postoperative recurrence and reoperation. On the other hand, it may avoid unwanted economic and psychological burdens for low-risk patients because of overtreatment.

With the development of precision medicine, the extent of initial surgery for MTC has become increasingly important with regard to the treatment and prognosis of patients. Therefore, we designed a retrospective study to assess the incidence of LLNM in MTC patients.

## Materials and Methods

The data we analyzed were extracted from two parts, one from the Surveillance, Epidemiology, and End Results (SEER) database between 2010 and 2017, and the other from our Department of Surgical Oncology, Hangzhou First People’s Hospital between 2009 and 2019.

### Data Selection From the SEER

The SEER*Stat software (version 8.3.8; https://www.seer.cancer.gov/seerstat) produced by the Surveillance Research Program and National Cancer Institute was applied to identify the MTC patients who had undergone surgical treatment (7). Inclusion criteria were the following (1): patients who were diagnosed from 2010 to 2017 with histopathology codes of the International Classification of Diseases for Oncology, 3rd edition (ICD-O-3), 8510/3, 8346/3, 8346/3, and 8346/3 (2); all patients treated with total thyroidectomy for MTC and were consecutively selected; and (3) patients who underwent central lymph node dissection (CLND) with lateral lymph node dissection. Exclusion criteria were the following (1): patients whose clinicopathological profiles were unclear (2); patients aged <18 and >85 years; and (3) patients with history of other malignant tumors. The clinicopathological profiles used the derived AJCC stage group (7th edition) and CSstage (https://cancerstaging.org).

### Data Selection From our Medical Center

We collected 35 patients with MTC confirmed by pathology in our medical center as the external testing set for this model. The pathological results of the external testing set were jointly diagnosed by two pathologists in our hospital. The histological type, extrathyroidal extension, tumor size, and number of the primary lesion were clearly defined. The lymph nodes are strictly based on the division of the surgeon. The calculation of the total lymph nodes includes the central compartment and the lateral compartment. If routine pathology is difficult to determine, immunohistochemistry will be used to assist in the diagnosis.

All patients in our medical center underwent open surgery. Thyroidectomy and cervical lymph node dissection were performed concurrently. For MTC tumors with a family history, extrathyroidal extension, bilateral or multifocal disease, and total thyroidectomy are advocated. Lymph node dissection includes CLND or LLND. Central lymph node dissection should be routinely performed during the initial operation. We performed preoperative neck mapping by both cervical ultrasonography and enhanced cervical computed tomography (CT). When lateral compartment lymph node metastasis was suspected by imaging or was defined by fine needle aspiration biopsy, patients underwent area II, III, IV, and Vb dissection. Patients underwent mediastinal lymph node dissection if mediastinal compartment lymph node metastasis was definitive.

### Statistical Analyses

Statistical analysis was performed by R Studio (Version 1.3.1093). Normally distributed data are expressed by mean ± standard deviation, and skewness data are expressed by interquartile range. The log‐rank test was used to compare the difference in the Kaplan–Meier (K–M) curves in recurrence and survival. The chi-square test and t-test were applied for univariate analysis. Then, multivariable logistic regression analysis was used to construct the predictive model based on the results, and a further nomogram was developed. Odds ratios (ORs) having 95% confidence interval (CI) were calculated. The prediction efficiency of the predictive model was assessed by area under the curve (AUC) and concordance index (C-index) and calibration curves in training set and testing set. Decision curve analysis (DCA) was also performed to determine the clinical utility of the predictive model by quantifying the net benefit at disparate threshold probabilities. *p* < 0.05 was statistically significant.

## Results

### Cervical Lymph Node Dissection and Metastasis

The median number of lymph nodes dissected was 22 (range,10-41 nodes), and the median number of positive lymph nodes present was 2 (range,0-11 nodes). In the training set, lymph node metastasis rate was 58.17%, lateral cervical lymph node metastasis rate was 44.02%.

### Demographics and Characteristics of Patients

There were 714 patients in the SEER program between 2010 and 2017 and 35 patients in the medical record of our medical center between 2009 and 2019 enrolled in this retrospective study. To build the clinical prediction model, all data were divided into three groups, which consisted of training set (n = 502, 70%), internal testing set (n = 212, 30%), external testing set (n = 35, our department). The specific clinical features of the patients in three datasets are summarized in **Table 1**.

**Table 1:** Clinicopathological characteristics of MTC patients.

Variable	Training set		Internal testing set		External testing set	
	LLNM (+) (n = 221)	LLNM (−)(n = 281)	LLNM (+) (n = 100)	LLNM (−) (n = 112)	LLNM (+) (n = 12)	LLNM (−) (n = 23)
Sex (male/female)*	131/90	78/203	64/36	40/72	9/3	7/16
Race (white/black/other)	191/18/12	232/25/24	84/11/5	93/3/16	0/0/12	0/0/23
Age (years)	53 ± 15	53 ± 14	52 ± 14	51 ± 15	55 ± 12	49 ± 11
Multifocal (unifocal/multifocal)*	121/100	208/73	56/44	81/31	12/0	21/2
Tumor size(mm)*	34 ± 20	23 ± 15	30 ± 18	22 ± 14	26 ± 14	13 ± 13
Extension (intrathyroidal/extrathyroidal)*	102/119	237/44	50/50	100/12	12/0	22/1
Nodes positive*	11 (6,19)	0 (0,1)	13 (7,23)	0(0,1.75)	2.5(0.25,4.75)	0 (0,0)
Distant metastasis (no/yes)*	189/32	280/1	89/11	112/0	12/0	23/0

*Indicating statistical significance.

LLNM, lateral lymph node metastasis; MTC, medullary thyroid carcinoma.

### Prognosis Review

**Figure 1:**
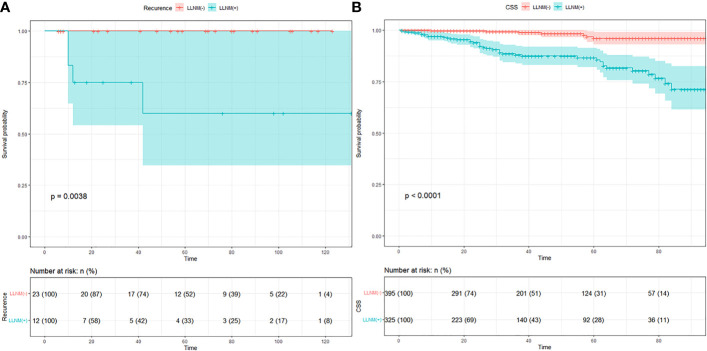
**(A)** K–M curve of recurrence in MTC patients of our medical center. **(B)** K–M curve of cancer specific survival in MTC patients of SEER data.

Follow-up data were obtained in all 749 patients. The mean follow-up time was 57 months with a follow-up time range of 6–131 months in our medical center. All patients were alive at follow-up, and there were four who had recurrence in the metastasis group. K–M curve indicated a significant difference in recurrence in **Figure 1A** (*p* = 0.0038). The mean follow-up time was 42 months with a follow-up time range of 1–95 months in the SEER database. There were 40 fatalities in the metastasis group and 7 fatalities in the non-metastasis group due to metastases. K–M curve indicated a significant difference in overall survival in **Figure 1B** (*p* < 0.001). The number of individuals are described in the risk table.

### Selection of Risk Factors for LLNM

A total of 221 in 502 cases of training set had positive lateral regional lymph nodes (44%). At univariate analysis, sex, multifocality, tumor size, extrathyroidal extension, and distant metastasis were found as the potential risk factors associated with LLNM in MTC patients (**Table 1**). The data for the tumor diameter were metric variable, so the cut-point of 38 mm was calculated for predicting lateral lymph node metastases. Multivariate logistic regression was further performed to screen for significant variables associated with LLNM. All of the variables were found to be significantly associated with LLNM (**Table 2**).

**Table 2:** Multivariate analysis results for LLNM in MTC patients.

Variable	Training set		Internal testing set	
	OR (95% CI)	*p* value	OR (95% CI)	*p* value
Sex	3.79 (2.61–5.51)	<0.001	3.2 (1.82–5.61)	<0.001
Multifocal	2.35 (1.62–3.43)	0.003	2.05 (1.16–3.64)	0.723
Extension	13.51 (7.57–24.1)	<0.001	17.2 (6.41–46.12)	<0.001
Tumor size	1.04 (1.03–1.05)	<0.001	1.03 (1.01,1.05)	0.066
M_stage	47.41 (6.42–349.91)	0.007	/	0.008

LLNM, lateral lymph node metastasis; MTC, medullary thyroid carcinoma; OR, odds ratio; CI, confidence interval.

### Nomogram Development and Validation for Prediction of LLNM in MTC Patients

**Figure 2:**
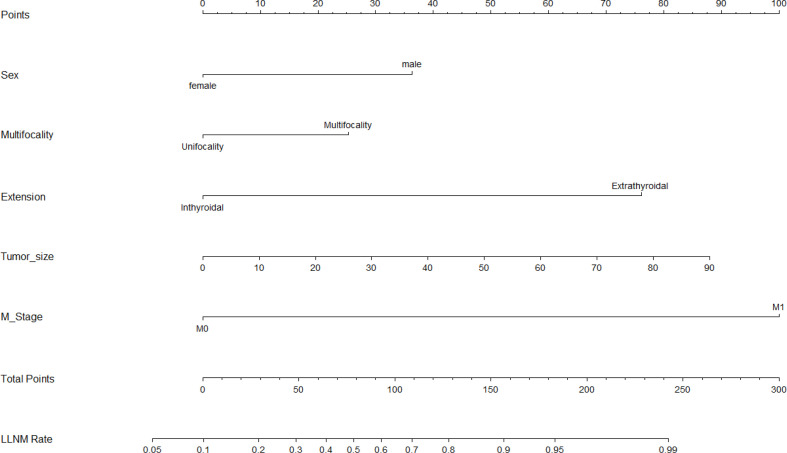
Nomogram for predicting LLNM in MTC patients. LLNM, lateral lymph node metastasis.

Based on the results that we analyzed in multivariate logistic regression model, five variables including sex, multifocality, tumor size, extrathyroidal extension, and distant metastasis were finally extracted to build an intuitive nomogram model for predicting the risk of LLNM in patients with MTC (**Figure 2**).

**Figure 3:**
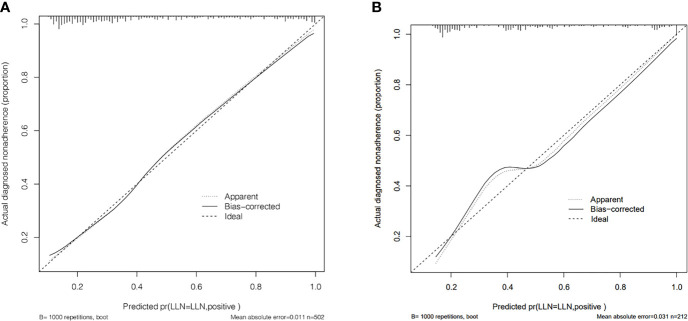
**(A)** Calibration curve of the nomogram for predicting LLNM in MTC patients for training set. **(B)** Calibration curve of the nomogram for predicting LLNM in MTC patients for the internal testing set.

**Figure 4:**
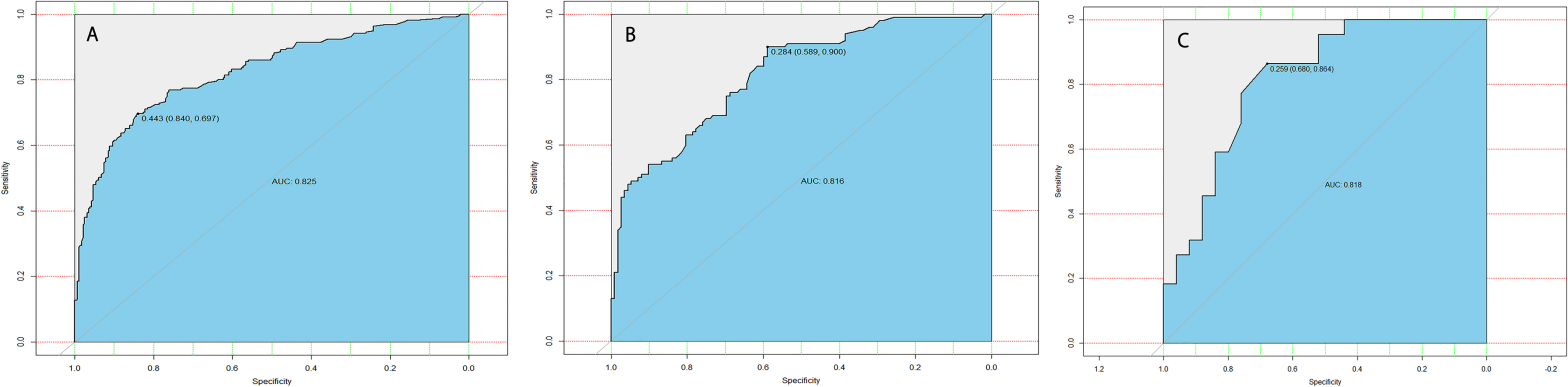
**(A)** ROC curve analysis to predict LLNM in MTC patients for training set. **(B)** ROC curve analysis to predict LLNM in MTC patients for the internal testing set. **(C)** ROC curve analysis to predict LLNM in MTC patients for the external testing set.

The calibration curve of the LLNM risk nomogram in MTC suggested great agreement in the training set and the internal testing set (**Figures 3A, B**). The C-index of the predictive model in the training set was 0.825 (95%CI, 0.697–0.840), and the internal testing set was 0.818, respectively. Area under the curve (AUC) was 0.825 for the training set, and the internal testing set was 0.818 (**Figures 4A, B**), which suggested the good prediction capability of the model.

Furthermore, 35 cases of MTC in our department were used for external testing set to estimate the practicability of the model. The results showed that AUC of the external testing set was 0.728, sensitivity was 68.0%, and specificity was 86.4% (**Figure 4C**), which suggested the good generalizability of the model.

**Figure 5:**
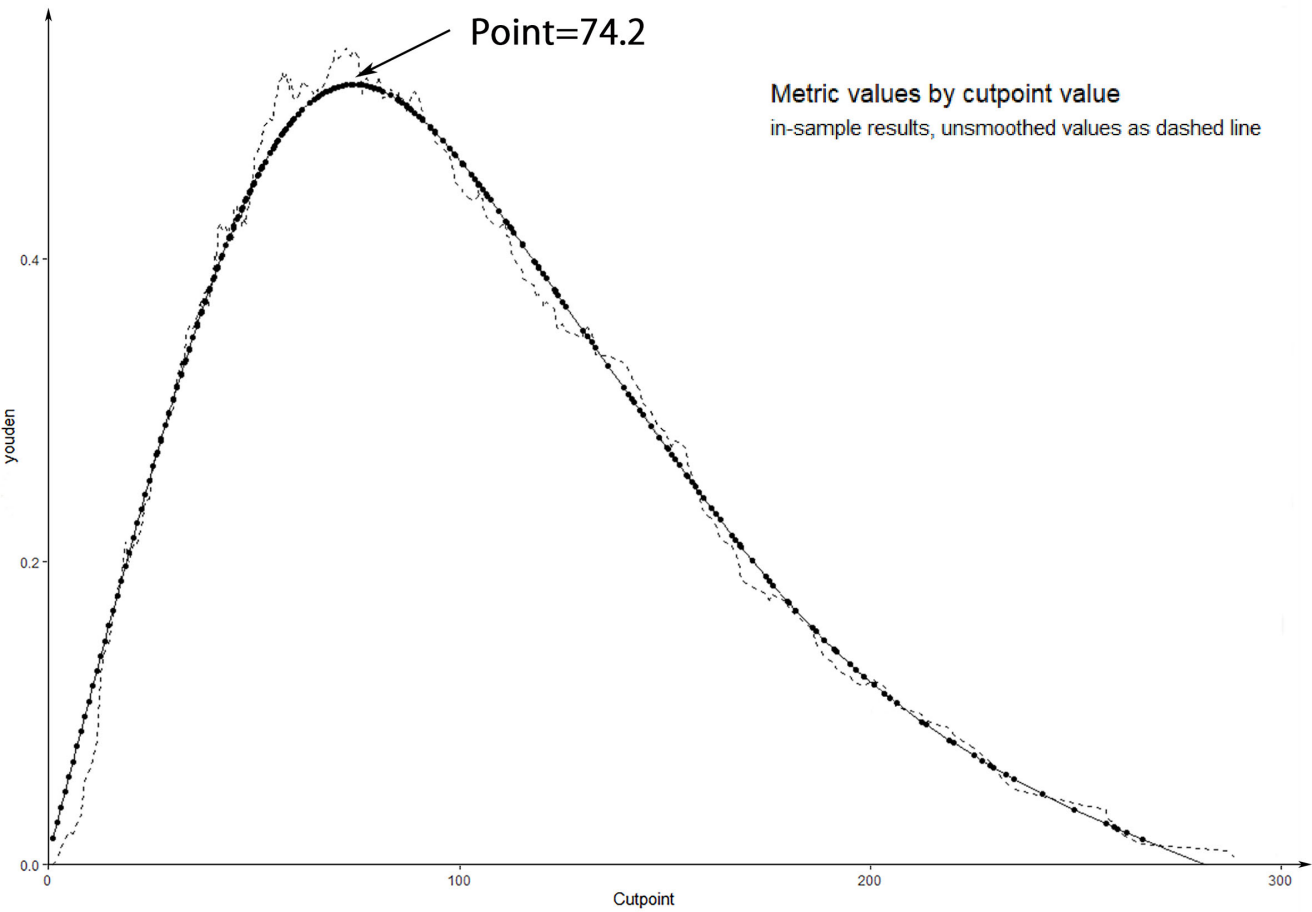
The curve of Youden and cut-point.

**Figure 6:**
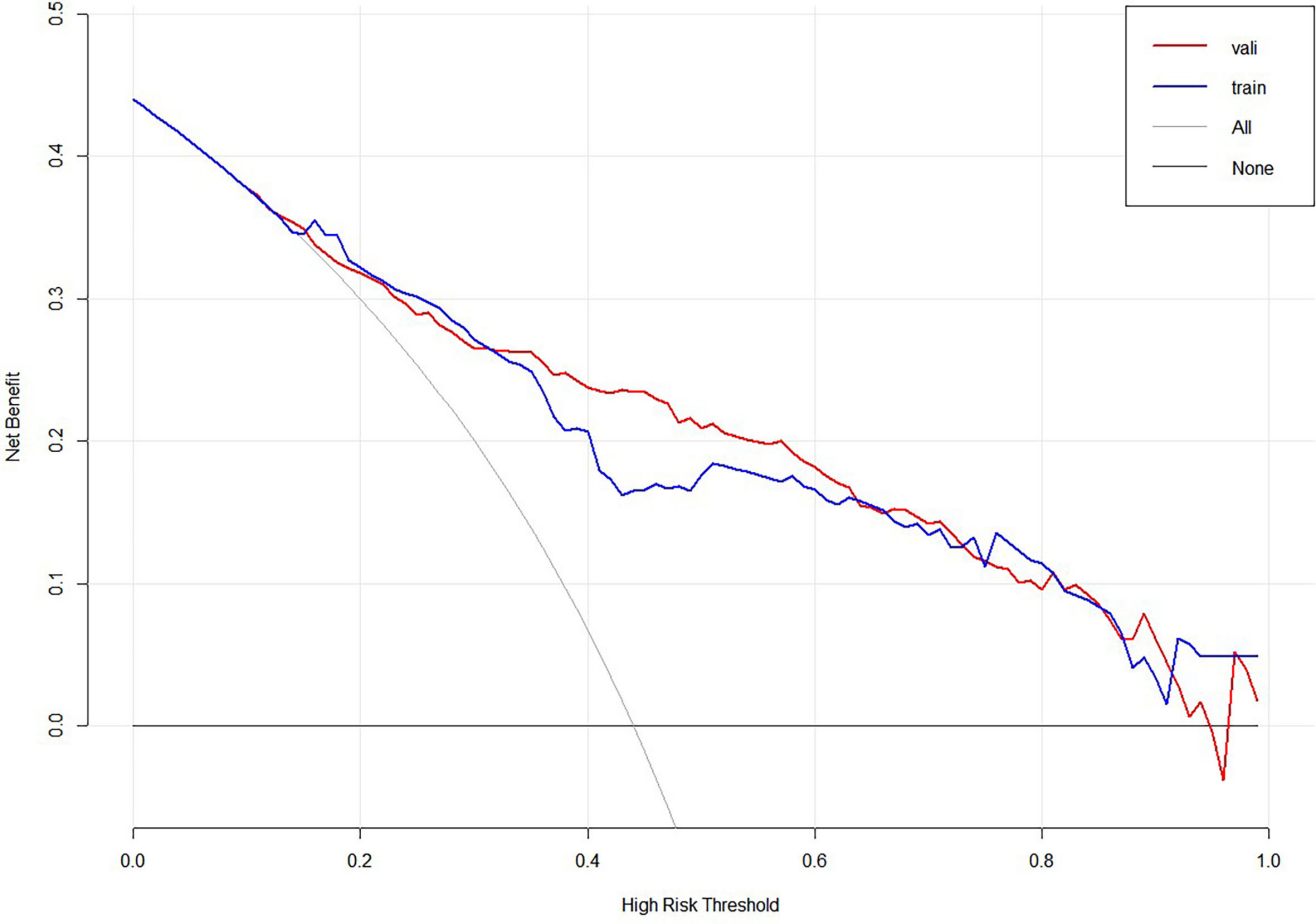
Decision curve analysis for LLNM in MTC patients.

To further evaluate the predictive capacity of the nomogram, patients with a risk score of 74.2 were the cut-point for predicting lateral lymph node metastases (Youden index = sensitivity + specificity −1; point = 74.2, **Figure 5**). The clinical value of the nomogram was assessed by DCA based on the net benefit and threshold probabilities. For example, when the value evaluated in the nomogram reaches a certain value, the probability of LLNM in patient I is denoted as Pi. When Pi reaches a certain threshold (denoted as Pt), it is considered positive. The ordinate of DCA is the net benefit (NB), which is defined as [true positives − false positives × Pt/(1 − Pt)]/sample number. The DCA curve can be obtained by plotting Pt from NB. We performed DCA, which showed that predicting the LLNM by applying the model would be better than having all patients or none patients treated by this model with a range of the threshold probability between >0.1 and <0.9 (**Figure 6**).

## Discussion

Medullary thyroid carcinoma is a group of carcinomas that intermediate between the differentiated thyroid carcinomas and anaplastic thyroid carcinoma, whose 10-year survival rate is approximately 80% (8, 9). This study revealed that patients with LLNM had worse recurrence rate and CSS compared with patients without LLNM. Approximately 29.4%–38% of MTC patients were accompanied with LLNM at the time of first diagnosis (10). In our study, the rate of LLNM was 46.64% in the SEER dataset and 34.23% in our department, which was consistent with the literature reports. Patients with LLNM predict a higher grade, higher aggression (tumor invasiveness), and increase in the risk of recurrence and death (11). Therefore, nodal metastasis assessment should be paid more attention to by surgeons.

In our study, male sex, tumor size >38 mm, multifocality, extrathyroidal extension, and distant metastasis in MTC patients were significant risk factors for predicting LLNM metastasis. This was consistent with the previous results reported by others. First, previous studies have found that extrathyroidal extension often reflects the tumor aggressiveness of MTC. A retrospective study showed that extrathyroidal extension was an independent risk factor for LLNM and recurrence (12). Our result showed that extrathyroidal extension was an independent risk factor, which is basically consistent with previous studies. Second, Fan et al. reported that tumor size >1 cm is associated with cervical lymph nodes; however only thyroid capsule invasion was identified as an independent risk factor for lateral lymph node involvement (13). Our study showed that tumor size and multifocal tumors are also important risk factors of LLNM, which was similar to the findings of Oh et al. (12). Demographic factors associated with LLNM are male sex and advanced age (14). However, age was not a risk factor of LLNM in our study, which is needed to be further verified by prospective researches of patients.

Lymph node metastasis is very common in MTC. Ultrasonography is the first imaging technique in the assessment of cervical lymph nodes metastasis of MTC patients preoperatively. CT exerts an influence on surgical strategy especially for patients with LLNM (15). It has been reported that ultrasonography has high specificity (85%–97.4%) but low sensitivity (36.7%–61%) in detecting cervical lymph node metastasis in MTC (16, 17). CT is more sensitive than ultrasonography for the assessment of the whole compartments of cervical lymph node metastasis. Although imaging plays a pivotal role in assessing lymph node metastasis, it still is challenging to accurately predict lymph node status by routinely used imaging modalities.

Nevertheless, at present, the scope of lymph node dissection in the treatment of MTC has been controversial because of the uncertainty of lymph node metastasis. ATA guidelines suggest that LLND is needed to be therapeutically performed on cN1 lateral cervical compartment, but the role of prophylactic dissection on cN0 lateral cervical compartment remains controversial (18). On the one hand, some scholars pointed out that the rate of occult metastasis in the cN0 lateral cervical compartment was also relatively high for patients with central lymph node metastasis. This high incidence of such occult metastases has a high postoperative recurrence and reoperation rate and a low biochemical cure rate. On the other hand, LLND did not confer a survival benefit for patients at early stage but lead to low quality of life even after cure. Some authors suggest that expanding surgical field increases the risk of injury to parathyroid glands and recurrent laryngeal nerves, without any demonstrable benefits in terms of long-term survival (4). Conzo et al. recommend that while the dissection of lateral cervical subzone is not straightforward, the complication rate is not higher for experienced thyroid surgeons (19). Liu et al. pointed out that the complication of LLND do correlate with the entire experience and surgical volume of the surgeon.

The multivariate logistic regression methods are trained using the SEER database to develop a preoperative model. In this model, the C-index and AUC value of the training set were both 0.825, and the calibration curve suggested that the actual probability of LLNM corresponded closely with the predicted probability of LLNM in MTC patients, which indicated relatively good discrimination ability (20). Furthermore, the external testing set showed that the AUC of this prediction model is 0.818. The predictive models can serve as complementary items for thyroid surgeons in choosing surgical treatment modality, and it is worthy of clinical promotion and popularization.

There are several limitations in the present study. First, the SEER database also lacks several important tumor maskers such as calcitonin and carcinoembryonic antigen, which benefits prediction of lateral lymph node metastasis in MTC. Second, another weakness is the lack of the number and size of the central cervical lymph nodes data. Third, the validation set is a single-center study with a small number of samples, so the applicability is limited to a certain extent, and further confirmation with a multicenter, large sample, and prospective clinical trial is needed in the future.

## Conclusion

MTC is a rare aggressive type of thyroid carcinoma, characterized by easy recurrence and early metastasis. Male sex, tumor size >38 mm, multifocality, extrathyroidal extension, and distant metastasis in MTC patients were significant risk factors for predicting LLNM. Our results may thus serve as a predictor for predicting LLNM in MTC and may perhaps also be referenced in preoperative discussions with patients regarding surgical methods.

## Data Availability Statement

The datasets presented in this study can be found in online repositories. The names of the repository/repositories and accession number(s) can be found below: https://seer.cancer.gov/.

## Ethics Statement

The studies involving human participants were reviewed and approved by the Ethics Committee of Hangzhou First People’s Hospital. The patients/participants provided their written informed consent to participate in this study.

## Author Contributions

T-HZ, L-QZ, and D-CL: conception and design. All authors contributed to acquisition, statistical analysis or interpretation of the data, and drafting of the manuscript. All authors contributed to the article and approved the submitted version.

## Funding

This work was supported by Science Research Program of Hangzhou (20180533B39), The Project of Medical Scientific and Technology Program in Hangzhou (grant number A20200432), and The Medical and Health Research Program of Zhejiang Province (2019RC240).

## Conflict of Interest

The authors declare that the research was conducted in the absence of any commercial or financial relationships that could be construed as a potential conflict of interest.

## Publisher’s Note

All claims expressed in this article are solely those of the authors and do not necessarily represent those of their affiliated organizations, or those of the publisher, the editors and the reviewers. Any product that may be evaluated in this article, or claim that may be made by its manufacturer, is not guaranteed or endorsed by the publisher.

## References

[1] Sung H Ferlay J Siegel RL Laversanne M Soerjomataram I Jemal A. Global Cancer Statistics 2020: GLOBOCAN Estimates of Incidence and Mortality Worldwide for 36 Cancers in 185 Countries. CA Cancer J Clin (2021) 71:209–49. doi: 10.3322/caac.21660 33538338

[2] Wells SA Jr Asa SL Dralle H Elisei R Evans DB Gagel RF. Revised American Thyroid Association Guidelines for the Management of Medullary Thyroid Carcinoma. Thyroid (2015) 25:567–610. doi: 10.1089/thy.2014.0335 25810047 PMC4490627

[3] Orloff LA Kuppersmith RB. American Thyroid Association's Central Neck Dissection Terminology and Classification for Thyroid Cancer Consensus Statement. Otolaryngol Head Neck Surg (2010) 142:4–5. doi: 10.1016/j.otohns.2009.11.013 20096215

[4] Spanheimer PM Ganly I Chou JF Capanu M Nigam A Ghossein RA. Prophylactic Lateral Neck Dissection for Medullary Thyroid Carcinoma Is Not Associated With Improved Survival. Ann Surg Oncol (2021) 28:6572–9. doi: 10.1245/s10434-021-09683-8 PMC8452790 33748897

[5] Lindsey SC Ganly I Palmer F Tuttle RM. Response to Initial Therapy Predicts Clinical Outcomes in Medullary Thyroid Cancer. Thyroid (2015) 25:242–9. doi: 10.1089/thy.2014.0277 25338223

[6] Opsahl EM Akslen LA Schlichting E Aas T Brauckhoff K Hagen AI. The Role of Calcitonin in Predicting the Extent of Surgery in Medullary Thyroid Carcinoma: A Nationwide Population-Based Study in Norway. Eur Thyroid J (2019) 8:159–66. doi: 10.1159/000499018 PMC6587193 31259158

[7] Warren JL Klabunde CN Schrag D Bach PB Riley GF. Overview of the SEER-Medicare Data: Content, Research Applications, and Generalizability to the United States Elderly Population. Med Care (2002) 40:IV–3-18. doi: 10.1097/00005650-200208001-00002 12187163

[8] Ceolin L Duval M Benini AF Ferreira CV Maia AL. Medullary Thyroid Carcinoma Beyond Surgery: Advances, Challenges, and Perspectives. Endocr Relat Cancer (2019) 26:R499–518. doi: 10.1530/ERC-18-0574 31252403

[9] Wang X Li C Huang L Shui CY Liu W Cai YC. Progression of Diagnosis and Treatment of Medullary Thyroid Carcinoma. Zhonghua Er Bi Yan Hou Tou Jing Wai Ke Za Zhi (2019) 54:306–10. doi: 10.3760/cma.j.issn.1673-0860.2019.04.015 30991785

[10] Zhao J Yang F Wei X Mao Y Mu J Zhao L. Ultrasound Features Value in the Diagnosis and Prognosis of Medullary Thyroid Carcinoma. Endocrine (2021) 72:727–34. doi: 10.1007/s12020-020-02510-2 33011881

[11] Maia AL Wajner SM Vargas CV. Advances and Controversies in the Management of Medullary Thyroid Carcinoma. Curr Opin Oncol (2017) 29:25–32. doi: 10.1097/CCO.0000000000000340 27792051

[12] Oh HS Kwon H Song E Jeon MJ Song DE Kim TY. Preoperative Clinical and Sonographic Predictors for Lateral Cervical Lymph Node Metastases in Sporadic Medullary Thyroid Carcinoma. Thyroid (2018) 28:362–8. doi: 10.1089/thy.2017.0514 29350102

[13] Fan W Xiao C Wu F. Analysis of Risk Factors for Cervical Lymph Node Metastases in Patients With Sporadic Medullary Thyroid Carcinoma. J Int Med Res (2018) 46:1982–9. doi: 10.1177/0300060518762684 PMC5991226 29569965

[14] Ito Y Miyauchi A Kihara M Higashiiyama T Fukushima M Miya A. Static Prognostic Factors and Appropriate Surgical Designs for Patients With Medullary Thyroid Carcinoma: The Second Report From a Single-Institution Study in Japan. World J Surg (2018) 42:3954–66. doi: 10.1007/s00268-018-4738-z PMC6244981 30051240

[15] Xing Z Qiu Y Yang Q Yu Y Liu J Fei Y. Thyroid Cancer Neck Lymph Nodes Metastasis: Meta-Analysis of US and CT Diagnosis. Eur J Radiol (2020) 129:109103. doi: 10.1016/j.ejrad.2020.109103 32574937

[16] Liu X Ouyang D Li H Zhang R Lv Y Yang A. Papillary Thyroid Cancer: Dual-Energy Spectral CT Quantitative Parameters for Preoperative Diagnosis of Metastasis to the Cervical Lymph Nodes. Radiology (2015) 275:167–76. doi: 10.1148/radiol.14140481 25521777

[17] Kim E Park JS Son KR Kim JH Jeon SJ Na DG. Preoperative Diagnosis of Cervical Metastatic Lymph Nodes in Papillary Thyroid Carcinoma: Comparison of Ultrasound, Computed Tomography, and Combined Ultrasound With Computed Tomography. Thyroid (2008) 18:411–8. doi: 10.1089/thy.2007.0269 18358074

[18] Asarkar A CB Nathan CO. What Is the Extent of Neck Dissection in Medullary Thyroid Carcinoma? Laryngoscope (2021) 131:458–9. doi: 10.1002/lary.28686 32311764

[19] Conzo G Docimo G Mauriello C Gambardella C Esposito D Cavallo F. The Current Status of Lymph Node Dissection in the Treatment of Papillary Thyroid Cancer. A Lit Review Clin Ter (2013) 164:e343–6. doi: 10.7417/CT.2013.1599 24045534

[20] Greiner M Pfeiffer D Smith RD. Principles and Practical Application of the Receiver-Operating Characteristic Analysis for Diagnostic Tests. Prev Vet Med (2000) 45:23–41. doi: 10.1016/S0167-5877(00)00115-X 10802332

